# CANCER PREVALENCE AND THE ROLE OF MULTIMORBIDITY AMONG OLDER ADULTS: USING NHANES 2011-2016

**DOI:** 10.1093/geroni/igz038.1721

**Published:** 2019-11-08

**Authors:** Fei Tang, Elizabeth Vasquez

**Affiliations:** 1 University at Albany - State University of New York, Albany, New York, United States; 2 University at Albany, SUNY, Albany, New York, United States

## Abstract

Cancer risk increases as age, understanding the potential risk factors of cancer is essential for cancer prevention. Biological and epidemiologic studies suggest relationships between individual chronic conditions and increased cancer risk. However, limited researches have analyzed the association between multimorbidity (simultaneous presentation of two or more chronic diseases) and cancer. The current study is aimed to evaluate whether having multimorbidity is associated with increased all-site and site-specific cancer prevalence among older adults. Data of 5,200 older adults aged 55 years and older who participated in the 2011-2016 National Health and Nutrition Examination Survey (NHANES) were included in the study. Single and multiple logistic regression models were used to evaluate the associations between multimorbidity and cancer. 3,623 (70%) individuals in our study were identified as having multimorbidity and 992 (19%) individuals were diagnosed with cancer. After adjusting for demographic covariates and smoking status, having multimorbidity was significantly associated with having all-site cancer (AOR: 1.57; 95% CI: 1.25 – 1.98) and lung cancer (AOR: 8.91; 95% CI: 1.51 - 52.73). Multimorbidity was associated with increased odds of having cancers among older adults. Our findings add to the evidence suggesting the potential relationships between multimorbidity and cancer. Future longitudinal studies are needed to examine the biological mechanisms and temporality of the association. If the association between multimorbidity and cancer is affirmed, it could have substantial implications in public health, as management of multiple chronic conditions could also advantage cancer prevention among older adults.

